# Using Murine Models to Investigate Tumor–Lymphoid Interactions: Spotlight on Chronic Lymphocytic Leukemia and Angioimmunoblastic T-Cell Lymphoma

**DOI:** 10.3389/fonc.2017.00086

**Published:** 2017-05-02

**Authors:** Tyler A. Herek, Christine E. Cutucache

**Affiliations:** ^1^Department of Biology, University of Nebraska at Omaha, Omaha, NE, USA

**Keywords:** lymphoma, chronic lymphocytic leukemia, angioimmunoblastic T-cell lymphoma, immune surveillance, tumor-induced immunosuppression

## Abstract

The role of the tumor microenvironment in leukemias and lymphomas is well established, yet the intricacies of how the malignant cells regulate and influence their non-malignant counterparts remain elusive. For example, chronic lymphocytic leukemia (CLL) is an expansion of malignant CD5^+^CD19^+^ B cells, yet the non-malignant T cells play just as large of a role in disease presentation and etiology. Herein, we review the dynamic tumor cell to lymphoid repertoire interactions found in two non-Hodgkin’s lymphoma subtypes: CLL and angioimmunoblastic T-cell lymphoma. We aim to highlight the pivot work done in the murine models which recapitulate these diseases and explore the insights that can be gained from studying the immuno-oncological regulation of non-malignant lymphoid counterparts.

## Introduction

The cancer immunosurveillance and immunoediting concepts posit that the immune system plays a protective role in tumor development through its ability to recognize and eradicate subclinical tumors (1, 2). These theories suggest an ever-shifting balance of tumor eradication and tumor growth wherein equilibrium can be lost and tumor growth can be promoted (3, 4). The promotion of tumor growth can be viewed as a consequence of the immune system as a function of microenvironmental interactions that have been shown to be vital for tumor development and survival (5). Therefore, with the immune system playing seemingly two separate, yet opposite, roles within immuno-oncology interactions it has garnered the “Janus-faced” moniker (3).

To understand the Janus-faced nature of tumor immunology is to understand the impact that a growing tumor has upon its microenvironment; by which we mean how tumors regulate their non-malignant counterparts. Perhaps it is not surprising the impact a tumor can have on the immune system when one considers all the working parts of the immune system function that function in an antitumor response (6). From NK cells lysing transformed clones, to presentation of tumor-associated antigens by macrophages and dendritic cells to activate T- and therefore B-cells, the immune system plays an integral role in producing and expanding tumor-specific responses (3).

**Figure 1:**
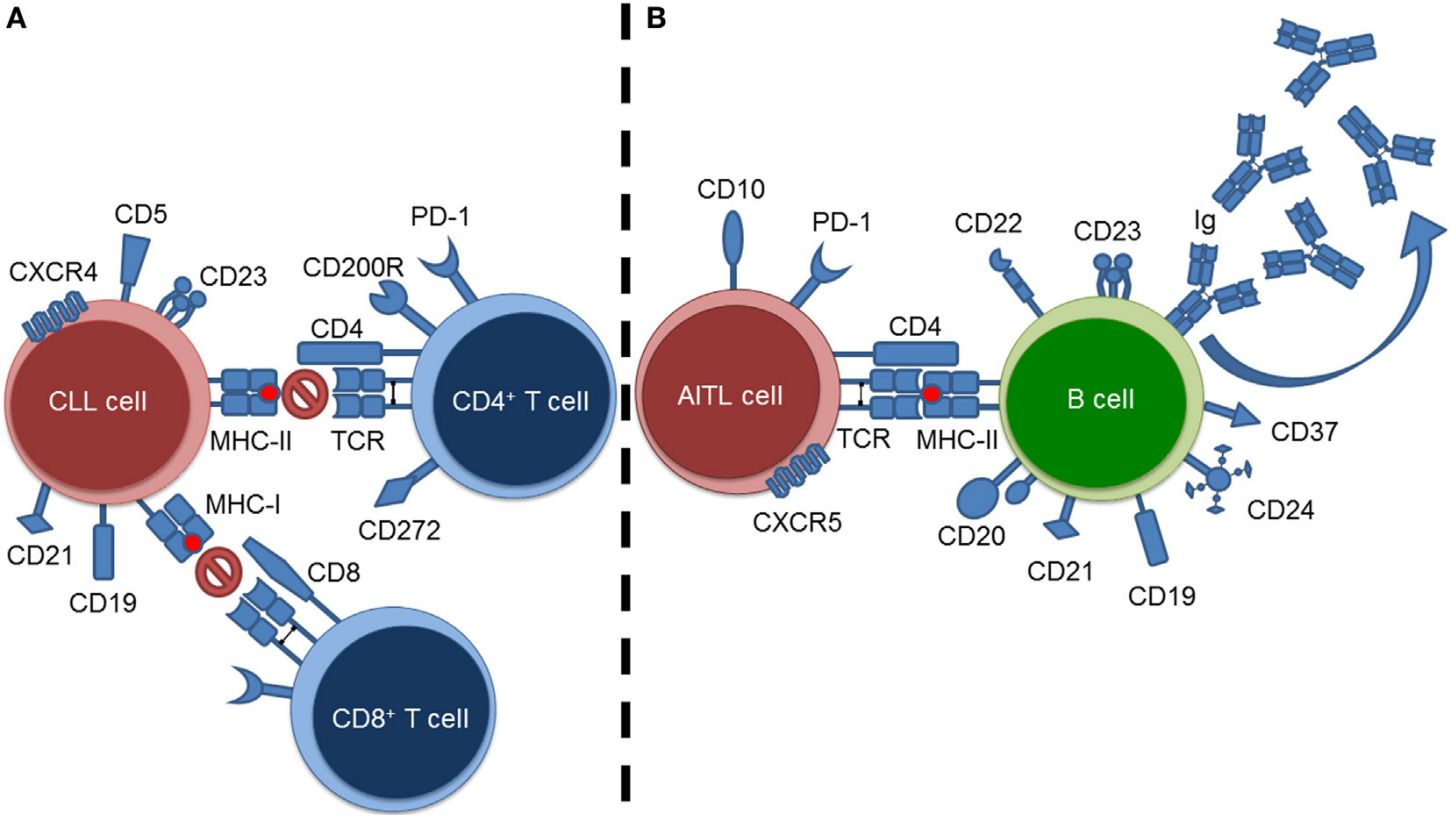
**Summary of tumor–lymphoid interactions in chronic lymphocytic leukemia (CLL) and angioimmunoblastic T-cell lymphoma (AITL)**. **(A)** CLL cell–resident T cell interactions exemplified by blockade of T-cell-mediated responses against the CLL cells. **(B)** AITL cell–resident B cell interactions exemplified by stimulation of B cells resulting in hypergammaglobulinemia.

Currently, the most effective method of studying the impact of a tumor microenvironment (TME) on a host immune system is through the use of murine models (7). Specifically, this would extend to transgenic murine models as they allow for the “natural” propagation of a clinical tumor within an immunocompetent host; most closely mirroring what is found in humans. In this review, we summarize and discuss the impact two distinct hematological malignancies, chronic lymphocytic leukemia (CLL) and angioimmunoblastic T-cell lymphoma (AITL), have upon their adaptive immunological microenvironmental counterparts (i.e., T cells and B cells, respectively) (Figure 1). Explicitly, we summarize how murine models play a role in elucidating the impact of a B-cell malignancy (i.e., CLL) on resident T cells, and the impact of a T-cell malignancy (i.e., AITL) on resident B cells. The aforementioned hematological malignancies were chosen as representative malignancies to study immuno-oncological interactions due to their intense microenvironmental components. As CLL is the most common adult leukemia, we are able rely on a wealth of publications and data from both human and murine investigations to summarize and discuss the above-mentioned interactions. AITL on-the-other-hand is a much rarer and less studied disease. Therefore, we will focus on what is currently known concerning AITL immuno-oncological interactions and put forward recommendations concerning AITL murine models and directions for future investigations.

## Chronic Lymphocytic Leukemia

Chronic lymphocytic leukemia is the most common adult leukemia characterized by the clonal expansion of malignant CD5^+^CD19^+^ B cells. The clinical presentation of CLL is heterogeneous, leading to both an indolent and aggressive form demarcated by well-defined prognostic markers (i.e., *IGVH* status, CD38 and ZAP70 expression, and chromosomal aberrations) (8–10).

Chronic lymphocytic leukemia is a malignancy highly dependent on its microenvironment illustrated by the fact that CLL cells readily undergo apoptosis *in vitro* without coculture of bone marrow stromal cells (BMSC) (11–13) or monocyte-derived nurse-like cells (NLCs) (13). While both BMSC and NLCs display similar recruitment of CLL cells through CXCL12–CXCR4 signaling (12–14), the mechanisms by which these compulsory stromal cells engage in cross talk with CLL cells differ. *In vitro*, CLL cells are attracted to BMSC and form a contact-dependent protective niche. While in contact with the BMSC, the CLL cells undergo a processes termed pseudoemperipolesis, which describes the migration of a fraction of the CLL cells beneath the BMSC (14–16). This migration results in the cross talk and bi-directional activation of both the BMSC and CLL cells leading to the upregulation of *TCL1* and FOS/JUN in the CLL cells (17). Conversely, NLC-dependent activation of CLL cells is characterized by enhanced CLL cell viability through NF-κB activation and the BAFF-/APRIL-binding pathways resulting in the expression of the anti-apoptotic protein MCL-1 by CLL cells for prolonged survival (18). Additionally, CLL cell activation through the B-cell receptor pathway is associated with increased secretion of CCL3 and CCL4 chemokines, allowing for the enhanced recruitment of accessory cells to the TME (19).

The progressive accumulation of CLL clones is primarily attributed to increased apoptotic resistance *via* exploitation by the above-described microenvironments (20); however, proliferative centers of CLL have been identified (21). These proliferation centers, termed pseudo-follicles, are composed of Ki-67^+^, Survivin^+^, p27^−^, Bcl-2^+^, CD23^Hi^ CLL cells and CD40L^+^ T cells (21–24). As a result, CD40 activation and IL-2/IL-10 signaling heighten CLL proliferation and upregulate IRF4 (25).

### The Impact of CLL on T Cells

The T cells of CLL patients display distinct expression profiles (26) and are hallmarked by an exhausted phenotype, attenuated immune synapse formation, decreased cytolitic activity, migratory impairments, and dysregulated Rho-GTPase signaling.

T-cell exhaustion is defined by T cells displaying an overexpression of inhibitory receptors, decreased effector function, attenuated cytokine production, and decreased cytolitic activity (27). CLL-T cells have been shown to upregulate the surface expression of PD-1, CD160, and CD244, indicative of an exhausted phenotype (28). Moreover, these markers are known to be highly expressed on effector T cells, indicating a skewing of the T-cell compartment of CLL patients to a more mature, effector differentiation, albeit with attenuated functionality. Further support for a skewed T-cell compartment comes from CD4^+^ CLL-T cells having decreased gene expression of the JNK and p38 MAPK pathway activators (*MINK, NFRKB, PIK3CB*) (26) suggesting a defect in Th1 differentiation as the aforementioned genes are crucial in the production of IFN-γ (29–31).

The immune synapse is a localization of receptors, costimulatory molecules, and adhesive anchors that allows for the engagement and recognition of antigen and activation by T cells (32). During the activation process, the cytoskeleton of the T cell undergoes intense rearrangements to better facilitate both contact and signaling through pseudopodia formation and cellular polarization. In essence, the T cell grasps the antigen-presenting cell (APC) forming what is called the immunological synapse (33). Structurally, the immune synapse is described as possessing three concentric rings called supramolecular activation clusters (SMACs) (34). The central supramolecular activation cluster houses the TCR molecules which are relocated from the periphery to be recycled, the peripheral SMAC is vital for maintaining adhesion between the cells and is primarily composed of the integrin, lymphocyte function-associated antigen 1 (LFA-1), and the distal SMAC is a clustering of F-actin. The engagement of the components of these SMACs influences the rearrangement of the cytoskeleton both by signaling relayed *via* the TCRs and through integrins such as LFA-1. For example, once LAT is phosphorylated *via* Zap70 following T-cell activation the Rho-family GTPase exchange factor vav guanine nucleotide exchange factor 1 (Vav1) is recruited to the synapse (35, 36). Following Vav1 activation, the small GTPases Ras-related C3 botulinum toxin substrate 1 (Rac1) and cell division control protein 42 homolog (Cdc42) bind GTP and activate actin nucleation promoting factors such as Wiskott–Aldrich syndrome protein (WASp) and Wasp-family verprolin-homologous protein 2 (WAVE2) which coordinate actin-related protein 2/3 (Arp2/3)-dependent polymerization of branched actin filaments (37–39). Following actin polymerization, the immunological synapse functions as a signal specifier acting to focus TCR signaling responses to ensure efficient cross talk with the bound APC. Dysregulation of the polymerization processes could therefore lead to inefficient effector function delivery through poor coordination of T-cell signaling or ineffectual delivery of signals to the APC.

Immune synapse formation, including both antigen presentation by CLL cells and the subsequent response by T cells, is attenuated in CLL patients. Phenotypically, immune synapse malformation of CLL-T cells is displayed as a decrease in T-cell–APC conjugation and F-actin polymerization, with further decrement in T-cell receptor, WASp, Dynamin-2, Lck, Cdc42, and Filamin-A recruitment to the synapse site (40, 41). Additionally, the inhibitory receptors CD200R, CD272, and CD279 are upregulated in CLL-T cells, and further impede immune synapse formation (28, 42). These aforementioned defects also contribute to decreased CD8^+^ T-cell cytolitic effector function, as granzyme B is inefficiently packaged and localized to the immune synapse in CLL CD8^+^ T cells (26, 28).

T cells from CLL patients display migratory defects through LFA-1 directed T-cell motility (36). This defect is concurrent with the previously described deficiency in cytoskeletal remodeling in CLL-T cells (26) and has been determined to be dependent upon LFA-1 conformation status. In terms of mRNA expression, there is no difference in gene expression of *LFA-1* between healthy T cells and CLL-T cells. Rather, CLL-T cells showed impaired migration due to the inefficiency of the low-affinity conformation of LFA-1 to bind intercellular adhesion molecule 1 causing an impediment in leukocyte rolling, arrest, firm adhesion, and diapedesis (43).

At the center of CLL-mediated T-cell impairments is the dysregulation of Rho-GTPase signaling in CLL-T cells. Rho-GTPases are members of the Ras superfamily of small GTP-binding proteins and play key roles in T-cell development, T-cell migration, and T-cell differentiation (44). This regulation is carried out through Rho-GTPase-mediated cytoskeletal rearrangements, as well as signaling through numerous pathways (45, 46). After coculture with CLL cells, T cells display lower active GTPase signaling for RhoA, Rac1, and Cdc42, as compared with coculture of T cells with healthy B cells (42). The dysregulation of these signaling molecules is also tied to defective LFA-1-mediated migration, where downregulated RhoA, Rac1, and potentiated Cdc42 are noted among CLL-T cells. Importantly, Cdc42 levels differed in CLL-T cell migration and stimulation assays and suggest that CLL-T cells do not lose all Rho-GTPase signaling potentials; however, they do not display identical signaling patterns as healthy T cells.

### Evidence from the Eμ-*TCL1* Model

T-cell impairment is a hallmark feature of CLL and is evident within the Eμ-*TCL1* murine model. Early investigations into the T-cell phenotype in Eμ-*TCL1* mice demonstrated decreased antigen-specific activation, suppressed mitogen initiated proliferation, and impaired idiotype specific CD8^+^ T cell-mediated CLL clone lysis (47). Gene expression profiling experiments supported the phenotypic differences, giving evidence to a dysregulation of the actin-cytoskeleton formation pathways, impaired F-actin polymerization, and immune synapse formation (47). Additionally, decreased expression of *CD28, BTLA, Nfrkb, Pi3k, Kfkb, Gadd45b*, and *Vav3* were observed.

Hofbauer and colleagues (48) were the first to report on the immunophenotype of Eμ-*TCL1* T-cells, finding an increase in the absolute numbers of T cells in the peripheral blood of leukemic mice, with the increased population consisting primarily of CD8^+^ T cells (~72%). Further, CLL-onset Eμ-*TCL1* mice experienced a shift between naïve and antigen experience T cells (as assessed by CD44 expression), presenting with a 48:48 CD4^+^ naïve:experienced ratio, compared to a 80:20 ratio in wild-type control mice. The CD8^+^ T cell ratio held a similar shift, with an 86:14 naïve:experienced ratio in Eμ-*TCL1* mice compared to a 70:27 ratio in wild-type controls (48). Importantly, this investigation brought a unique insight into the pathogenesis of CLL by establishing the engraftment of CLL spleenocytes within an immunocompetent host. This adoptive transfer model decreased the latency of CLL onset (83 days post-transfer compared to 247 days of Eμ-*TCL1* development) and fully recapitulated the abovementioned skewing of the T-cell compartment. These data implicate the transferred CLL clones as being directly responsible for the onset of disease progression and changes to the T-cell compartment (48).

Recently, extensive work by the Gribben lab sought to delineate the mechanism behind the PD-1/PD-L1 signaling axis in T cells from Eμ-*TCL1* mice, as well as provide important evidence for use of PD-L1 checkpoint blockade therapy (49, 50). Importantly, these studies lend microenvironmental data to the known role of PD-1/PD-L1 signaling in human CLL (49). As CLL developed in the Eμ-*TCL1* mice, there was a significant correlation in the reduction of the percentage of CD3^+^, CD3^+^CD4^+^ T cells, and an expansion of CD3^+^CD8^+^ T cells in the spleen. This finding is in agreement with Hofbauer et al. (48) of a decrease in the CD4^+^/CD8^+^ ratio in Eμ-*TCL1* mice for the spleen, peripheral blood, and lymph node microenvironments. Further, CD3^+^CD8^+^CD44^−^CD62L^+^ naïve cells were lost with CLL development, with a shift toward CD44^+^ antigen-experienced T cells in the spleen, peripheral blood, lymph node, and bone marrow microenvironments. These antigen-experienced CD44^+^ T cells held higher proliferation indices, as evidenced by increased Ki-67^+^ ratios in Eμ-*TCL1* mice and adoptive transfer recipients (spleenocytes transferred into 3-month Eμ-*TCL1* mice).

Consistent with aging, both Eμ-*TCL1* and wild-type mice developed CD3^+^CD8^+^PD-1^+^ T cells, with higher absolute numbers found in Eμ-*TCL1* and adoptive transfer mice (49). Investigation into the differential function of PD-1^+^ subsets in Eμ-*TCL1* mice revealed an enrichment within the PD-1^+^ population for cytotoxic cells (as assessed by CD107a^+^) with increased proliferation ascribed to both PD-1^high^ and PD-1^low^ subsets, with the PD-1^high^ population having greater EdU incorporation compared to PD-1^low^. Finally, immune synapse assays indicated the PD-1^high^ population to form smaller synapses when compared to the PD-1^low^ population in Eμ-*TCL1* T cells, suggesting the PD-1^high^ phenotype to contribute more toward T-cell impairment (49).

A causal role for the PD-1/PD-L1 signaling axis impairing clone-specific immune responses in Eμ-*TCL1* mice has been supported by the work of both McClanahan and colleagues (50) and Gassner et al. (51). α-PD-L1 blockade administered to Eμ-*TCL1* mice resulted in the effective control of CLL development, concurrent with reduction in both spleen sizes and weights (50). Treatment of adoptively transferred CLL tumor cells with either recombinant PD-1 or anti-CD274 resulted in a significant reduction of transferred tumor cells at both 2- and 24-h time points in peripheral blood (both time points), as well as in the spleen, lymph nodes, and lungs (24-h time point) (51). Antibody blockade restored the relative median frequencies of CD3^+^, CD4^+^, and CD8^+^ T cells, while normalizing the CD4^+^/CD8^+^ ratio (50). This restoration was observed in conjunction with a reduced shift toward the CD44^+^ phenotype seen in the peripheral blood, bone marrow, and spleen.

## Angioimmunoblastic T-Cell Lymphoma

Angioimmunoblastic T-cell lymphoma is the second most common subtype of mature T-cell lymphoma, known to originate by the pathogenic clonal expansion of T-follicular helper (Tfh) cells and concordant immune dysfunction (52, 53). AITL is an aggressive disease, with a median survival <3 years post-diagnosis and 10–30% of patients alive at 5 years post-diagnosis (54, 55). The poor prognosis is partially due to the highly dysregulated immune system of AITL patients, as most patients succumb to infectious complications rather than the disease itself (54, 55).

The changes AITL imparts upon the lymph node microenvironment are considered a consequence of the cellular origins of the disease. AITL patients display aberrant lymph node architecture, including: hyperplasia of the follicular dendritic meshwork (53), proliferation of high-endothelial venules, thickened extra follicular meshworks, and neoplastic infiltrate (56). The neoplastic cells can be medium sized with round, slightly irregular nuclei featuring abundant clear cytoplasm, with clustering around the high-endothelial venules (57, 58). The neoplastic cells themselves may only account for 5–30% of the tumor (59), and most of the clinical manifestations of the disease (i.e., lymphadenopathy, hepatosplenomegaly, bone marrow involvement) represent complications arising from a compromised immune system (60, 61).

As stated, the cell-of-origin for AITL is the Tfh cell. In healthy conditions, these cells are formed from high affinity interactions with B cells though a T-cell receptor:antigen-dependent manner (62). Tfh cells are located within germinal centers and assist with the production of high affinity antibodies by B cells, as well as plasma cell differentiation, primarily through secretion of IL-21 and IL-4 (63, 64). These cells express high levels of ICOS and CXCR5 and can be immunophenotypically described as CD4^+^, CD8^−^, PD-1^+^, CD10^+^, BCL6^+^, and CXCL13^+^ cells (59, 65, 66). As with many mature T-cell lymphomas, AITL cells also exhibit defective expression of CD5 and CD7 (65), with further clonal aberrations detected in ~90% of cases (58, 67–69). The gene signature of AITL is described as very closely related to that of Tfh cells, with upregulations of *VEGF, PDGFRα*, B-cell and follicular dendritic cell genes, chemokines, extracellular matrix components, and vascular biology-associated genes (70, 71). Such findings are in accordance with the changes to the lymph node architecture and the common involvement of B cells in AITL disease progression.

### The Impact of AITL on B-Cells

Due to a compromised immune system, in conjunction with dysregulation of normal immune effector functions, there is a high proclivity for developing secondary B-cell malignancies among AITL patients (72). These secondary malignancies commonly present as either composite lymphomas of diffuse large B-cell lymphoma + AITL (73, 74) or polyclonal populations of immunoblasts or plasma cells (75, 76). The development of these secondary malignancies suggests that the malignant T cells still hold the effector capacity to stimulate B-cell proliferation and antibody production and indicate a relationship between the developments of the two separate disease phenotypes (77, 78). Evidence supporting the above hypothesis includes: development of hypergammaglobulinemia, gene signature analyses demonstrating an upregulation of genes for B-cell activation and/or receptor signaling (CD22, CD20, CD21, CD23, CD24, CD37) (79), and histological evaluation of germinal centers displaying SH2D1A staining, a protein involved in bi-directional activation of T and B cells (80).

### Murine Models That Recapitulate the Defining Characteristics of AITL

Unlike the Eμ-*TCL1* mice for CLL, there is no current consensus murine model for AITL. Currently, three genetic models most faithfully recapitulate the defining disease characteristics of AITL and how they impact resident B cells will be discussed below.

#### *Tet2^gt/gt^* Model of AITL

The ten–eleven translocation 2 (TET2) gene has been found to be mutated in multiple myeloid malignancies (81–83) as well as a wide range of AITL and PTCL-NOS cases (84, 85). A loss of function mutation in TET2 is associated with aberrant methylation processes involving the conversion of methylcytosine to hydroxymethylcytosine, a common epigenetic marker in cancer pathogenesis (86).

While previous studies of *Tet2* mutations have been conducted (87–89), Muto and colleagues were the first to describe the resultant disease in terms of its relation to the development of a T-cell lymphoma with follicular helper T-cell-like features (90). After backcrossing *Tet2* gene trap mice onto the C57BL/6 background and aging for 40–60 weeks, *Tet2^gt/gt^* mice had no differences in complete blood counts or proportions of total T- or B-cell populations in comparison to wild-type or heterozygous controls. The *Tet2^gt/gt^* mice presented with splenomegaly and preserved follicle structures and an enlargement of germinal centers in some mice. Flow cytometry analyses revealed an increase in the absolute #’s of T- and B-cell populations, despite harboring no differences in the proportions of the overall populations. Further analyses displayed an increase in the proportions of Tfh populations in the *Tet2^gt/gt^* mice, namely: CD4^+^CD44^+^PD-1^+^ and CD4^+^PD-1^+^Cxcr5^+^ Tfh cells.

In five of seven mice aged greater than 60 weeks (median age: 67 weeks), Muto and colleagues (90) observed the development of increased splenomegaly, multiple swollen lymph nodes, and liver and lung nodules in *Tet2^gt/gt^* mice. Histological analysis demonstrated the follicular structures of the spleen to be no longer apparent due to the infiltration of large polymorphic cells with irregular nuclei and characterized as CD4^+^PD-1^+^Cxcr5^+^ Tfh cells. These T cells were determined to be clonal in nature, with distinct rearrangements of TCR Vβ/Jβ2. Not observed histologically was proliferation of high-endothelial venules, nor eosinophil infiltration. Then, >60-week mice presented with increased proportions of CD4^+^ T-cell fractions and smaller B220^+^ B-cell fractions, with the T-cell population mostly composed of CD44^+^PD-1^+^ cells. Of note, there was no difference in serum Ig levels found in *Tet2^gt/gt^* mice compared to controls.

Consistent with the observation that the malignant cells in *Tet2^gt/gt^* mice were of Tfh origin, microarray analysis of isolated splenic CD4^+^ T cells from *Tet2^gt/gt^* mice compared to control mice found the *Tet2^gt/gt^* T cells to be enriched for Tfh genes. Notable genes found to be highly expressed in the *Tet2^gt/gt^* mice include: *Bcl6, cMaf, PD-1, Icos*, and *Cxcr5*. Control CD4^+^ T cells were enriched for genes involved in Th1/Th2 differentiation by comparison, suggesting the downregulation of these genes is necessary for the adoption of the Tfh phenotype. Sanger sequencing experiments indicated that none of the concurrent mutations identified within human AITL (e.g., *Flt3, Npm1, Dmnt3a, Idh2, Rhoa*) were found in the *Tet2^gt/gt^* mice. Investigation into the differential methylation patterns resulting from reduced *Tet2* function revealed disruption of the distribution of methylcytosine and hydroxymethylcytosine in transcriptional start sites, gene body regions, and CpG islands. Specifically, *Bcl6* was identified as having aberrant methylation suggesting this as a mechanism for increased *Bcl6* expansion and the induction of the outgrowth of Tfh-like cells (90).

#### Heterozygous *Roquin^San^* Model of AITL

The *sanroquin* mouse strain bears a homozygous point mutation in the *Roquin/Rc3hl* gene (91), designated as *Roquin^san^*. This point mutation results in increased binding affinity between ROQUIN and *Icos* mRNA resulting in the decreased decay rate of the transcript and overall upregulation of gene product (92). The upregulation of *Icos* in these mice results in the expansion of Tfh cells driving a disease progression equivocal to a systemic lupus-like disease phenotype—a similar phenotype is observed in *Roquin^san/+^* mice but in the absence of systemic autoimmunity (91). However, mice heterozygous for the *Roquin^san^* allele (*Roquin^san/+^*) as described in Ellyard et al. (93) do not develop systemic autoimmunity but instead present with asymmetric lymphadenopathy. *Roquin^san/+^* mice had a complete absence of generalized lymphadenopathy seen in homozygous mice by 8 weeks, but instead, 53% developed one to four enlarged lymph nodes. The resultant lymphadenopathy was deemed non-lethal and caused an increase in the cellularity of the affected lymph nodes by 50- to 150-fold. A higher prevalence in females (65%) compared to males (41%) was noted for the development of the tumor-bearing lymph nodes. Histological examination of tumor nodes in *Roquin^san/+^* mice demonstrated an effacement of nodal architecture and vascularization, as well as infiltration of small-to-medium sized T cells and large PAX5^+^ B cells, but no expansion of the follicular dendritic cell network. An increase in F4/80^+^ macrophages was observed with prominent vascularization and sinusoidal dilation. Reactive B blasts were observed in interfollicular areas along with a proliferative and polymorphic T-cell infiltrate, small clusters of mature plasma cells, and some rosette formation between T cells and large blasts. Tumor lymph nodes appeared to have no bearing on un-affected lymph nodes in tumor-bearing *Roquin^san/+^* mice, as un-affected nodes displayed typical morphologies with the exception of an increase in mature plasma cells found within interfollicular areas. All *Roquin^san/+^* mice presented with increased spleen weight compared to *Roquin^+/+^* mice regardless of tumor incidence.

Concurrent with the highly affected lymph node environment of the *Roquin^san/+^* mice, increased serum IgG is observed consistent with the development of hypergammaglobulinemia at 15 weeks of age. While both *Roquin^san/+^* tumor-bearing and non-tumor-bearing mice show a significant increase in IgG production compared to *Roquin^+/+^* mice, tumor-bearing *Roquin^san/+^* mice harbor an additional 1.5-fold increase above non-tumor-bearing *Roquin^san/+^* mice. A finding further corroborated by flow cytometry analysis revealing an increased B-cell fraction in the tumor-bearing lymph nodes of *Roquin^san/+^* mice in comparison to normal lymph nodes from both *Roquin^+/+^* and *Roquin^san/+^* mice. Clonality assessments identified a clonal peak of IgH in only 1 of 15 cases, in contrast to clonal arrangements in 12 of 15 cases for TCR-β.

#### Swiss Jim Lambert (SJL) Model of AITL

Swiss Jim Lambert mice have been studied extensively as a model of lymphoproliferative malignancies. In these mice, the germinal center B cells express an endogenous mouse mammary tumor virus (mtv-29) superantigen (vSAg) known stimulate and activate CD4^+^ T cells which in turn drive B cell proliferation (94, 95). Through this dual activation paradigm, heterogeneous lymphoproliferative disorders develop in >90% of mice (96). Recent studies have characterized the heterogeneity of SJL lymphomas finding similarities to non-Hodgkin lymphomas and moving away from the classical reticulum cell sarcoma diagnosis (97, 98).

Jain and colleagues (99) are the first to describe the similarities between RCS and AITL. In an analysis of 80 SJL mice, ~44% developed classical SJL disease [i.e., Non-Hodgkin lymphoma and/or reticulum cell sarcoma (100)]. In SJL disease-diagnosed mice ranging from 2 to 24 months of age, flow cytometry demonstrated an expansion of Tfh cell frequencies (CD4^+^CXCR5^+^ICOS^+^PD-1^+^) in the spleens of SJL mice compared to C57BL/6 controls. Additionally, increased frequencies of splenic GC B cells (B220^+^FAS^+^GL-7^+^) were also found in SJL mice compared to C57BL/6 controls. Microarray analysis of a time course of SJL spleens (mice aged 6 weeks, 6 months, 12 months) revealed a progressive upregulation of genes involved in Tfh signaling, namely: *Il21, Il10, Ccl8*, and *Ccl12*. Knockout experiments investigating the impact of IL-21-mediated signaling on the SJL mice established that *Il21r^−/−^* SJL mice showed significant reductions in frequencies of total CD4^+^ T cells and Tfh cells in 9.5- to 12-month mice. A finding concurrent with reduced frequencies of GC B cells seen in 3- to 12-month *Il21r^−/−^* SJL compared to SJL WT controls (99). This finding verifies the IL-21 signaling as the etiology of SJL-diseased mice, and re-affirms SJL disease as Tfh-based as IL-21 is considered the major effector cytokine for Tfh cells (101).

Diagnosed SJL-diseased mice underwent age-related changes in splenic and lymph node architecture as assessed by peanut agglutinin (PNA) reactivity. Younger mice had well preserved follicular structures, but by 12 months had uniformly enlarged structures with uneven PNA reactivity. Some mice were noted as having highly enlarged follicles that did not contain germinal centers. The uncontrolled expansion of these follicles leads to the white pulp compressing the red pulp in the spleen.

Higher sera IgG2b protein levels were found in SJL mice compared to C57BL/6 controls. *Il21r^−/−^* mice exhibited lower sera IgG2b sera levels compared to wild-type SJL mice, implicating the expanded Tfh cell population as a driver of the development of hypergammaglobulinemia. *Igh* and *Tcrb* genes from spleens of primary lymphoma or SJL-diagnosed mice were sequenced to assess the presence or absence of clonal populations within the diseased mice. All sequenced *Tcrb* clones contained identical V gene sequences, albeit with different J gene sequences. For *Igh* sequences, high somatic hypermutation was evident across all malignant samples with overall identities <95%.

## Conclusion

The Eμ-*TCL1* mouse is a powerful model for CLL with a highly analogous microenvironment to that of the human disease (102). Reviewed herein, we see the powerful effect a B-cell malignancy can have on its T-cell counterparts. In CLL, there is a skewing of the T-cell compartment toward antigen-experienced CD8^+^ cytotoxic T cells. These cells further express exhaustion markers (e.g., PD-1) that demonstrate their inability to effectively control malignant clones. In this scenario, we see that a tumor-specific immune response has been stymied by the malignancy itself, allowing for continued tumor growth and dissemination. Support for this observation is seen in that adoption of the exhausted and skewed T-cell phenotype is dependent on the presence of CLL clones (48), and α-PD-1 blockade therapies are successful in restoring the protective capability of the immune system (50, 51). These results demonstrate the potential of treatments aimed at restoring immune equilibrium to help combat tumors *in vivo* and give further credence to the necessity and power of murine models to support bench-to-bedside investigations.

While there is no current consensus murine model for AITL, we have reviewed the three most prominent proposals to-date. Of these, both the heterozygous *Roquin^san^* and SJL models appear most suited to be used in future studies modeling AITL microenvironmental interactions. Importantly, both of these models faithfully recapitulate defining disease characteristics of AITL, including: Tfh expansion, hypergammaglobulinemia, effacement of lymphoid architecture, clonal populations, and increased high-endothelial vascularization. It stands to reason that future investigations should be aimed not only at additional model validations and Tfh-based characterization but also focus on the B-cell involvement of AITL that could be key to disease pathogenesis.

Taken together, both murine models for CLL and AITL effectively recapitulate the *in vivo* expansion of key immune cells. Studies on CLL that have included both cell types in their investigations have provided exceptional clinical insight to modulate immune competence and provide treatment for CLL (including through immunomodulation therapy). Consequently, as seen in CLL, therapies targeted at the lymphoid counterpart of AITL (i.e., B cells) could hold high efficacy and aide in the understanding of tumor microenvironmental manipulation of resident B cells.

## Author Contributions

TH and CC cowrote the paper in its entirety.

## Conflict of Interest Statement

The authors declare that the research was conducted in the absence of any commercial or financial relationships that could be construed as a potential conflict of interest. The reviewers, CS and RJ, and handling editor declared their shared affiliation, and the handling editor states that the process nevertheless met the standards of a fair and objective review.
